# Does uneven geographic distribution of urologists effect bladder and prostate cancers mortality? National health insurance data in Korea from 2007–2011

**DOI:** 10.18632/oncotarget.18036

**Published:** 2017-05-20

**Authors:** Jae Heon Kim, Hwa Yeon Sun, Hyun Jung Kim, Young Myoung Ko, Dong-Il Chun, Jae Young Park

**Affiliations:** ^1^ Department of Urology, Soonchunhyang University Hospital, Soonchuhyang University Medical College, Seoul, Korea; ^2^ Department of Preventive Medicine, College of Medicine, Korea University, Seoul, Korea; ^3^ Department of Industrial and Management Engineering, Pohang University of Science and Technology, Pohang, Korea; ^4^ Department of Orthopaedics, Soonchunhyang University Hospital, Soonchuhyang University Medical College, Seoul, Korea; ^5^ Department of Urology, Korea University Ansan Hospital, Korea University College of Medicine, Ansan, Korea

**Keywords:** urologist density, bladder cancer, prostate cancer, mortality

## Abstract

The relationship between distribution of urologists and mortality of bladder and prostate cancers has not been clearly established. The aim of this study was to investigate the relationship between uneven distribution of urologists and urologic cancer specific mortality at country level. Data from the National Health Insurance Service and National Statistical Office in Korea from 2007 to 2011 were analyzed in this ecological study. Univariate and multivariable regression analyses were performed to determine risk factors for age standardized mortality rates (ASMR) of bladder and prostate cancers. Linear regression analysis showed a markedly (*p* < 0.001) uneven distribution of urologists between metropolitan and non-metropolitan areas. There was no significant difference in cancer specific ASMRs for either bladder cancer or prostate cancer. Univariate analysis after adjusting for time showed that country area, urologist density, and income were significant factors affecting bladder cancer incidence (*p* < 0.001, *p* = 0.013, and *p* < 0.001, respectively). It also showed that the number of training hospitals was a significant factor for prostate cancer incidence (*p* = 0.002). Although country area showed borderline significance (*p* = 0.056) for ASMR of bladder cancer, urologist density was not related to ASMR of bladder cancer or prostate cancer. Although there was a marked difference in urologist density between metropolitan and non-metropolitan areas for these years analyzed, mortality rates of bladder and prostate cancers were not significantly affected by country area or urologist density.

## INTRODUCTION

Although there have been great developments in medical services, uneven geographic distribution of clinicians is becoming one critical problem in the perspective of community health [1]. An uneven distribution of physicians in rural areas has been generally recognized [2] to be associated not only with treatment of common diseases, but also with treatment of specific cancers [3].

Physicians can be categorized to two types according to their expected roles: primary care physicians and specialists. It has been reported that mortalities from cancers, including urological cancers and gastrointestinal tract cancers, are affected by an uneven density of urologists and general surgeons [4–6].

Recently, Odisho et al. [6] have performed a national ecological study and reported that mortality rates of kidney, bladder, and prostate cancers in rural areas are affected by urologist density. However, there are conflicting data about this issue [7, 8]. Other reports have indicated that urologist density is not directly correlated with improved mortality rates [9, 10]. This phenomenon is related to the so-called “plateau phenomenon”, which reported not only for urological cancers, but also for other cancers [11].

The uneven distribution of physicians, including urologists, could be more significant because aging is progressing rapidly in the society. In a generally aging society, aging of physicians are also expected. Moreover, South Korea is one of several countries with an extremely rapid rate of aging. An uneven distribution of primary physicians and urologists for these years analyzed in this study has been documented in our previous study [12].

The aim of this study was to determine the impact of types of country areas, including metropolitan area and rural area, and urologist density after adjustment for years on bladder cancer and prostate cancer incidence and mortality. Although many studies about this issue have been reported in the US, this is the first study on this issue in Asia. Due to the aging society, public health policy has to be schemed with limited budget in the future. Therefore, it is important to emphasize cost-effectiveness when treating diseases besides proper density and distribution of doctors. Results of this study could provide useful information to prepare proper strategy for future policies in public health.

## RESULTS

### Basic characteristics

Basic characteristics of two county units (metropolitan area and non-metropolitan area) are shown in Table 1. ASMR1 and ASMR2 showed no significant differences between metropolitan area and non-metropolitan area. Incidence of bladder cancer in non-metropolitan area was significantly higher than that in metropolitan area in 2007 and 2008 (*p* = 0.032 and *p* = 0.005, respectively). Incidence of prostate cancer in non-metropolitan area was also significantly higher than that in metropolitan area in 2007 and 2009 (*p* = 0.035 and *p* = 0.036, respectively).

**Table 1:** Basic characteristics of metropolitan and non-metropolitan areas

		Bladder cancer			Prostate cancer		
		Metropolitan	Non-metropolitan	*p*-value^†^	Metropolitan	Non-metropolitan	*p*-value
ASMR1	2007	2.1	1.9	0.498	2.3	2.1	0.611
	2008	2.2	1.9	0.429	2.5	2.3	0.490
	2009	2.0	1.8	0.250	2.8	2.4	0.205
	2010	2.0	2.2	0.242	2.7	2.6	0.565
	2011	2.3	2.3	0.776	2.8	2.7	0.371
ASMR2	2007	3.2	2.9	0.339	4.7	4.3	0.539
	2008	3.3	2.9	0.390	5.0	4.5	0.459
	2009	3.1	2.7	0.243	5.7	4.8	0.210
	2010	3.0	3.4	0.225	5.6	5.5	0.706
	2011	3.4	3.5	0.862	5.8	5.5	0.547
Incidence	2007	5.7	7.2	0.032	18.1	24.4	0.035
	2008	5.8	7.5	0.005	22.2	29.0	0.064
	2009	6.1	7.2	0.068	25.4	33.5	0.036
	2010	6.4	7.4	0.152	26.8	35.4	0.050
	2011	6.3	7.8	0.096	81.1	92.8	0.774
Doctor density	2007	159.5	120.3	0.021	159.5	120.3	0.021
	2008	166.6	127.7	0.035	166.6	127.7	0.035
	2009	173.2	133.1	0.036	173.2	133.1	0.036
	2010	170.2	130.8	0.045	170.2	130.8	0.045
	2011	170.2	130.8	0.045	170.2	130.8	0.045
Urologist density	2007	4.1	3.1	0.007	4.1	3.1	0.007
	2008	4.1	3.3	0.019	4.1	3.3	0.019
	2009	4.4	3.4	0.013	4.4	3.4	0.013
	2010	4.5	3.5	0.011	4.5	3.5	0.011
	2011	4.6	3.6	0.020	4.6	3.6	0.020
No. of training hospital	2007	7.9	3.7	0.370	7.9	3.7	0.370
	2008	7.9	3.7	0.370	7.9	3.7	0.370
	2009	7.9	3.7	0.370	7.9	3.7	0.370
	2010	7.9	3.7	0.370	7.9	3.7	0.370
	2011	7.9	3.7	0.370	7.9	3.7	0.370
Income	2007	12193.1	10811.2	0.087	12193.1	10811.2	0.087
	2008	12730.1	11378.4	0.094	12730.1	11378.4	0.094
	2009	13173.3	12028.3	0.129	13173.3	12028.3	0.129
	2010	13915.9	12300.0	0.042	13915.9	12300.0	0.042
	2011	15033.0	13185.0	0.040	15033.0	13185.0	0.040
Temp	2007	14.2	13.3	0.153	14.2	13.3	0.153
	2008	13.9	13.0	0.147	13.9	13.0	0.147
	2009	13.9	13.0	0.176	13.9	13.0	0.176
	2010	13.5	12.6	0.192	13.5	12.6	0.192
	2011	13.3	12.4	0.203	13.3	12.4	0.203

*P* value by *t*-test.

### Annual change of doctor density and urologist density

**Figure 1:**
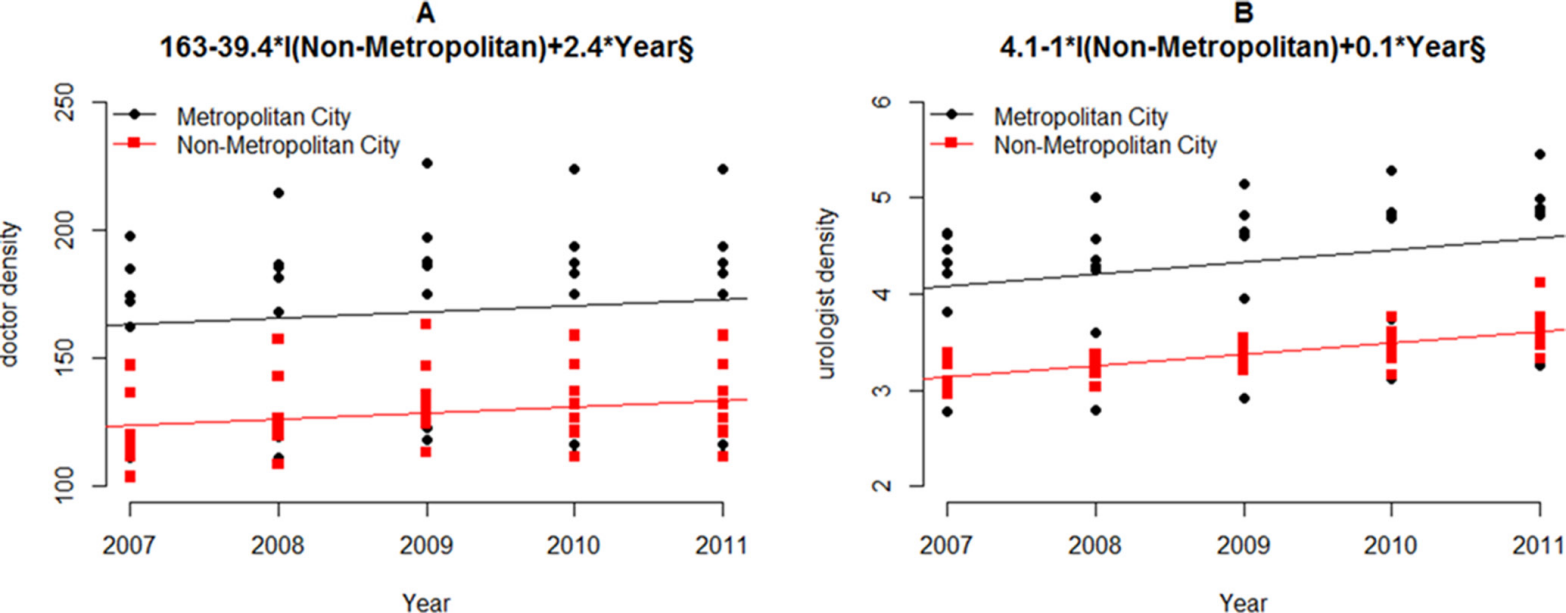
Annual changes of doctor density and urologist density according to years

Annual changes of doctor density and urologist density in metropolitan and non-metropolitan areas are shown in Table 2 and Figure 1. Both doctor density and urologist density showed were higher in metropolitan areas compared to non-metropolitan areas (both *p* < 0.001). However, after adjusting years with reference year of 2007, only urologist density was higher in metropolitan area compared to that in non-metropolitan area (*p* = 0.002).

**Table 2:** Annual changes of doctor density and urologist density according to metropolitan and non-metropolitan areas

		**beta**	**se**	**Lower**	**Upper**	***p*-value**
Doctor	intercept	163.1	6.1	150.9	175.3	< 0.001*
	area(non-metropolitan)	–39.4	6.0	–51.3	–27.5	< 0.001*
	year^†^	2.4	2.1	–1.7	6.6	0.249
Urologist	intercept	4.1	0.1	3.9	4.3	< 0.001*
	area(non-metropolitan)	–1.0	0.1	–1.2	–0.7	< 0.001*
	year^†^	0.1	0.0	0.0	0.2	0.002*

^†^Year of 2007 was used as reference year.

*Statistically significant.

### Trends of incidence and mortality of bladder cancer and prostate cancer

Trends of incidence, ASMR1 and ASMR2, for the period of 2007 to 2011 are shown in Table 3. The incidence of bladder cancer showed no significantly increasing pattern in either metropolitan area or non-metropolitan area (*p* = 0.182 and *p* = 0.378, respectively). However, mortality of bladder cancer showed significantly increasing pattern in both metropolitan and non-metropolitan areas (*p* = 0.006 and *p* = 0.001, respectively). For bladder cancer, ASMR1 showed significantly increasing trend in both metropolitan and non-metropolitan areas (*p* = 0.028 and *p* = 0.003, respectively). However, ASMR2 showed significantly increasing pattern only in non-metropolitan area (*p* = 0.001). Estimates of ASMR1 and ASMR2 in non-metropolitan area were higher than those in metropolitan area. For prostate cancer, both ASMR1 and ASMR2 showed no significant increase in metropolitan areas. However, in non-metropolitan areas, both ASMR1 and ASMR2 showed significantly increasing patterns (*p* = 0.014 and *p* = 0.009, respectively).

**Table 3:** Linear trend of incidence and mortality of bladder and prostate cancers

			**Estimate**	**Std. Error**	***p*-value**
Bladder					
Metropolitan	ASMR1	year	0.137	0.060	0.028*
	ASMR2	year	0.274	0.140	0.059
	Incidence	year	0.193	0.142	0.182
Non-metropolitan	ASMR1	year	0.145	0.046	0.003*
	ASMR2	year	0.324	0.094	0.001*
	Incidence	year	0.124	0.140	0.378
Prostate					
Metropolitan	ASMR1	year	0.045	0.057	0.437
	ASMR2	year	0.022	0.096	0.820
	Incidence	year	13.077	4.414	0.006*
Non-metropolitan	ASMR1	year	0.101	0.040	0.014*
	ASMR2	year	0.178	0.065	0.009*
	Incidence	year	14.322	4.009	0.001*

*Statistically significant.

### Univariate analysis for cancer incidence, ASMR1, and ASMR2

**Figure 2:**
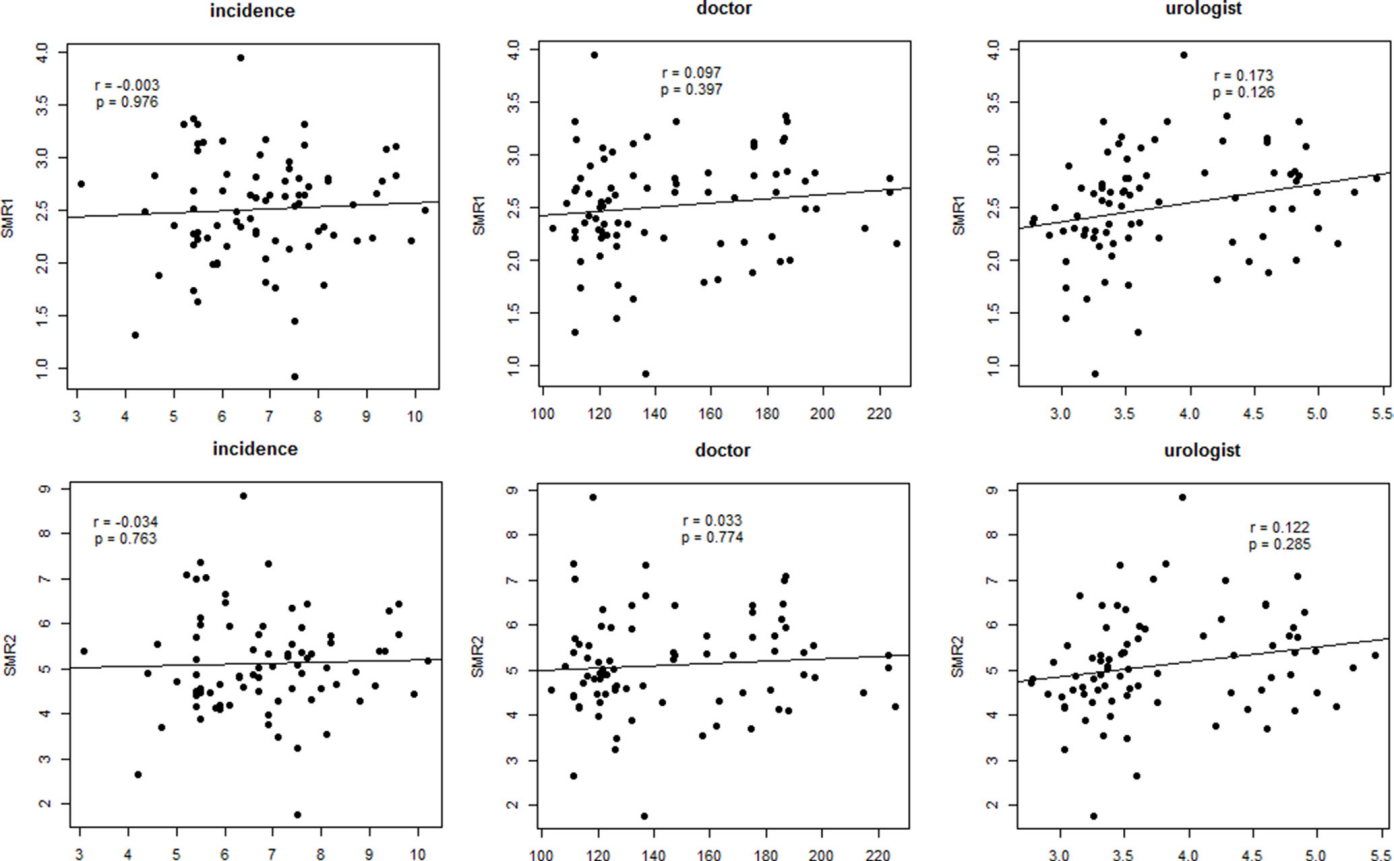
Partial correlation analysis between cancer mortality rate or cancer incidence and doctor density or urologist density in bladder cancer

**Figure 3:**
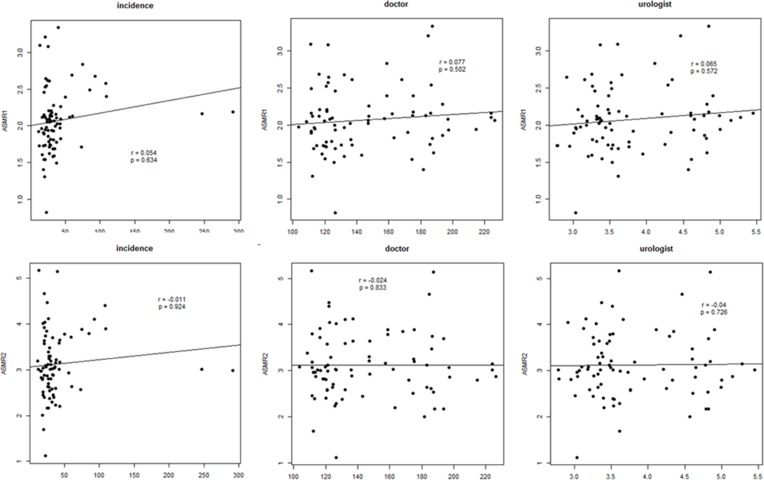
Partial correlation analysis between cancer mortality rate or cancer incidence and doctor density or urologist density in prostate cancer

Univariate analysis after adjusting for time showed that geographic area, urologist density, and income were significant factors affecting bladder cancer incidence (*p* < 0.001, *p* = 0.013, and *p* < 0.001, respectively). It also showed that the number of training hospitals was a significant factor for prostate cancer incidence (*p* = 0.002) (Table 4). Temperature was the only significant factor affecting ASMR of bladder cancer, although geographic area showed a borderline significance for ASMR1 and ASMR2 of bladder cancer (*p* = 0.056 and *p* = 0.060, respectively). Doctor density and urologist density showed no significant correlation with either ASMR1 or ASMR2 (Figures 2 and 3).

**Table 4:** Univariate analysis for cancer incidence, ASMR1, and ASMR2

	**Bladder cancer**				**Prostate cancer**			
	**beta**	**95% CI**		***p*-value**	**beta**	**95% CI**		***p*-value**
		**Lower**	**Upper**			**Lower**	**Upper**	
Incidence								
Area (non-metropolitan)	1.384	0.820	1.949	0.000*	8.283	–8.465	25.032	0.328
No. of training hospital	–0.030	–0.071	0.010	0.143	1.617	0.612	2.623	0.002*
Doctor density	–0.009	–0.019	0.000	0.055	0.085	–0.171	0.341	0.510
Urologist density	–0.588	–1.050	–0.127	0.013*	4.219	–8.272	16.710	0.503
Income	0.000	–0.001	0.000	0.000*	0.001	–0.005	0.008	0.650
Temp	–0.153	–0.398	0.092	0.217	–5.594	–11.918	0.730	0.082
ASMR1								
Area (non-metropolitan)	–0.202	–0.409	0.005	0.056	–0.094	–0.283	0.095	0.324
No. of training hospital	0.000	–0.013	0.013	0.997	–0.001	–0.013	0.011	0.900
Doctor density	0.001	–0.002	0.005	0.397	0.001	–0.002	0.004	0.502
Urologist density	0.120	–0.035	0.276	0.126	0.040	–0.101	0.181	0.572
Income	0.000	0.000	0.000	0.248	0.000	0.000	0.000	0.700
Temp	0.019	–0.062	0.100	0.645	0.026	–0.046	0.099	0.473
Incidence	–0.001	–0.076	0.073	0.976	0.001	–0.002	0.003	0.634
ASMR2								
Area (non-metropolitan)	–0.438	–0.894	0.018	0.060	–0.117	–0.434	0.200	0.466
No. of training hospital	–0.013	–0.043	0.016	0.368	–0.010	–0.030	0.010	0.343
Doctor density	0.001	–0.006	0.008	0.774	–0.001	–0.005	0.004	0.833
Urologist density	0.186	–0.158	0.530	0.285	–0.042	–0.278	0.195	0.726
Income	0.000	0.000	0.000	0.140	0.000	0.000	0.000	0.551
Temp	0.211	0.039	0.383	0.017*	0.081	–0.039	0.201	0.184
Incidence	–0.025	–0.189	0.139	0.763	0.000	–0.004	0.004	0.924

*p*-value adjusted by year;

*Statistically significant.

### Multivariable analysis for bladder cancer incidence

Since there was no significant factor for ASMR, a multivariable analysis was conducted only for bladder cancer incidence. Results are shown in Table 5. Geographic area was the only significant factor for bladder cancer incidence, with a higher (*p* = 0.001) incidence in non-metropolitan area compared to that in metropolitan area. For prostate cancer, training hospital was the only significant factor for its incidence (*p* = 0.005).

**Table 5:** Multivariable analysis for incidence of bladder and prostate cancers

	**Bladder cancer**				**Prostate cancer**			
	**beta**	**95% CI**		***p*-value**	**beta**	**95% CI**		***p*-value**
		**Lower**	**Upper**			**Lower**	**Upper**	
Area (non-metropolitan)	1.401	0.558	2.243	0.001				
No. of training hospital					1.487	0.460	2.514	0.005
Doctor density								
Urologist density	0.384	–0.216	0.985	0.206				
Income	0.000	0.000	0.000	0.055				
Temp					–3.677	–9.865	2.512	0.240

*p*-value adjusted by year;

*Statistically significant.

## DISCUSSION

Establishing an adequate doctor workforce and its proper distribution is becoming an issue of great importance considering the increasing demand for cancer care [13, 14]. However, correlation between specific physician or surgeon density and specific cancer mortality has been inconsistent. Main reasons for such inconsistence include not considering regional variations during analysis [4, 6, 15] and “plateau phenomenon” [6]. These reasons could also be applied to urologic cancers.

Several recent studies have reported that there is no correlation between uneven distribution of urologists and mortality of bladder and prostate cancers [9, 10]. Fry et al. [9] have reported that urologist density is related to mortality rate of kidney and renal pelvis cancer, but not to that of bladder or prostate cancer. Yao et al. [10] have performed an updated ecological study using exploratory spatial analysis and found that there is an inconsistent relationship between urologist density and mortality rate of prostate cancer according to regional differences.

In our study, although the total duration of 5 years was not long, doctor density and urologist density did not correlate with mortality rate of bladder cancer or prostate cancer. These results were obtained after considering difference in incidence of both cancers by year. The incidence of bladder cancer did not show significantly increasing pattern. However, the incidence of prostate cancer showed significantly increasing pattern in both metropolitan and non-metropolitan areas. For bladder cancer, there was an increasing pattern of cancer mortality in rural areas (Table 3). For prostate cancer, there was an increasing pattern of cancer mortality in both geographic areas. However, the increasing pattern was more prominent in non-metropolitan area (Table 3). These results provided valuable information that unevenly increasing patterns of cancer mortality in both cancers were not affected by uneven distributions of doctor density or urologist density. The phenomenon of a more prominent increasing pattern of cancer mortality could be explained by age factors. Although age standardization process was accomplished with ASMR1 and ASMR2, age was still a powerful impacting factor. Although mortality rate before age standardization was not shown in this study, there was a marked difference in mortality rates between metropolitan and non-metropolitan areas. In addition to age factor, there are other possible reasons for the non-correlation between urologist density and mortality rate of bladder cancer or prostate cancer.

First, biological factors of each cancer might have contributed to such non-correlation. It is well-known that the general mortality rate dominates over prostate cancer specific mortality, especially in indolent prostate cancers. For bladder cancer, initial intensive treatment may not guarantee a more favorable survival outcome due to its biological feature of frequent recurrence [8]. Our results revealed that incidence and mortality of bladder cancer showed significant or borderline significant difference according to regional distribution. However, they showed no significant difference according to doctor density or urologist density. This suggests that biological factor of bladder cancer could be manifested differently according to circumstance.

Second, there are many confounding factors in determining a correlation between urologist density and bladder or prostate cancer mortality. Those factors consist of disease characteristics (tumor stage, tumor characteristics, and comorbidities) and socioeconomic factors (insurance status, annual income, education degree, and religion) [10]. Colli et al. [16] have reported that all cancer mortality is consistently correlated with income. Frye et al. [9] have also reported that uneven house income is an independent risk factor for mortality of kidney and bladder cancers. Clegg et al. [7] have shown that prostate cancer mortality is related to family income.

Third, screening might have affected the non-correlation. Urologists perform diagnostic and treatment processes. Hence, earlier detection of cancers due to higher urologist density in both metropolitan and non-metropolitan areas might have produced better mortality outcome. Aneja et al. [13] have reported that radiation oncologists might have greater potential role than urologists because they are not involved in the screening process. However, their study also showed the limitation of plateau effect which provided marginal benefit. Such plateau effect has also been documented in other fields on doctor density [17, 18]. Mortality rate of urologic cancer could be decreased by earlier detection of cancer. However, this does not mean that patients with earlier detection are treated optimally due to higher urologist density with well-distribution.

Fourth, the distance between rural area and metropolitan area or the distance between home and hospital call medical accessibility is also an impacting factor for cancer mortality. Moreover, medical expense in Korea is relatively low compared to that in other countries. Therefore, ‘plateau phenomenon’ from US or other western countries might need to be interpreted differently from this study. Moreover, as shown in our previous study [12], average urologist density in Korea is larger than that in US. In kidney cancers, a greater distance to a large volume hospital with available technical treatment such as partial nephrectomy is related to overall mortality [9]. Hence, distance to a metropolitan area where there are larger volume hospitals with more specialists is related to cancer mortality [19–21]. However, this could not be an issue in Korea because medical accessibility is excellent due to developed transport system and relatively small distances. Such excellent medical accessibility might have resulted in the no-correlation between urologist density and mortality rates of bladder and prostate cancers.

Fifth, although it is a minor factor, difference in statistical method may play a role in inconsistent results among previous studies. All previous studies and the current study conducted linear or logistic regression models to investigate the relationship. However, Yao et al. [10] adopted geographically weighted regression model to control spatial non-stationarity in doctor residences.

Finally, racial factor might have affected this non-correlation. Most previous ecological studies about doctor supply and cancer mortality have been carried out in the US. This study is the first study carried out in Asia. Considering racial disparities including percentage of black people which might have affected prostate cancer mortality, our study might have also been affected by race factor. Therefore, results of this study need to be interpreted differently than those US studies.

Although this study is the first study in Asia, there remains several limitations. First, this study has a short duration for mortality outcome (from 2007 to 2011). However, this study approached mortality for all those years. It was not a cross-sectional study.

Second, this study categorized metropolitan and non-metropolitan areas. This resulted in fewer county units than other categorizations such as metropolitan, general city, and rural areas [12]. However, previous studies had sampling limitations in that most of them did not include rural areas [6, 9]. It is important to include rare events in rural areas because the main focus of most ecological studies is to determine proper distribution of doctors and hospital volumes. Without excluding rural area, we categorized county units as metropolitan and non-metropolitan areas.

Third, due to the nature of ecological studies, this study did not include individual characteristics or covariate data. Moreover, data were not generated by patient units. Therefore, characteristics of data were indirect. Hence, caution is needed when interpreting results from ecological studies.

Lastly, although our study provides useful evidence to determine proper urologist density, more evidence is needed to incorporate Korea specific situations. Medical accessibility is relatively excellent in Korea owing to developed transportation and low medical expenses. This could be a rationale that Korea does not need the same urologist density as required by US. However, evaluating proper urologist density is not simple because we not only have to consider factors for providing optimal treatment for urologic cancers, but also have to consider factors involved in in screening and providing palliation treatment for urologic cancers. Future studies are warranted to examine the potential of urologist density on the entire quantity of urologic cancers from screening to mortality and examine the inter-relationship between urologist density and cancer mortality considering individual patient levels, health care resources, and sociodemographic components [10]. In addition, comparative ecological studies are needed regarding health services provided for urologic cancers after adjusting for individual social features in developing countries and developed countries.

In summary, an uneven distribution of doctors and urologists in metropolitan and non-metropolitan areas existed in Korea in the period of 2007 to 2011. A marked increase in mortality rate of bladder and prostate cancers was found in non-metropolitan areas during the analysis period. However, age standardized mortality rates of bladder and prostate cancer were not affected by the uneven distribution of doctors and urologists by years. Further studies are needed to demonstrate the adequacy of doctors and urologists to predict favorable prostate and bladder cancer mortality considering cost-effectiveness.

## MATERIALS AND METHODS

### Study design

This was an ecological study using national representative data in Korea from 2007 to 2011. Metropolitan and non-metropolitan areas were geographic units for analysis. A total of 16 county units, including 7 metropolitan units and 9 non-metropolitan units, were adopted to cover the absence of mortality rate information for rural areas. Moreover, our previous study has revealed that differences in doctor and urologist densities between metropolitan areas and non-metropolitan areas (non-metropolitan cities or rural areas) are evident [12]. These 16 county units are mainly based on the definition by public administration which establishes demographic, geographic, and environmental characteristics of country levels. The geographic unit of the country was defined as metropolitan area and non-metropolitan area established by the Ministry of Security and Public Administration. In Korea, the Ministry of Security and Public Administration currently lists a total of 163 county units at national level. However, we divided the total country into 16 units (7 metropolitan cities and 9 non-metropolitan areas) because most rural areas did not have detailed mortality data. Moreover, some rural areas have been merged into general cities. According to the Ministry of Security and Public Administration, a metropolitan city was defined as county units with more than 1,000,000 population. A non-metropolitan area was defined when the sum of county units has more than 50,000 in population and less than 50,000 in population in each county unit. Most of this study design and methods have been described in a previous report [12].

### Definition of variables including doctor density, urologist density, and covariates

Numbers of doctors and urologists in each county unit were obtained from the National Health Insurance Service and the Ministry for Health, Welfare and Family Affairs. This is different from the US where the American Medical Association (AMA) Masterfile provides the number of physicians [22]. Urologist density was defined as the number of urologists per 100,000 individuals at county level. Doctor density was defined as the number of doctors per 100,000 individuals at county level. Doctors include all doctors who have certification as a medical doctor, including primary physicians and specialists. Population data was obtained from the Population Census Division, National Statistical Office of Korea. Total productivity sum of each county unit and individual income data were obtained from the Survey Management Bureau, National Statistical Office of Korea.

Local temperatures were obtained from the National Atmospheric Administration of Korea. South Korea is located in the southern portion of the Korean Peninsula, which extends about 1,100 km (680 mi) from the Asian mainland. The country, including all its islands, lies between latitudes of 33° and 39°N with longitudes 124° to 130°E. Its total area is 100,188 square kilometers (http://www.ngii.go.kr/kor/board/view.do?rbsIdx=103&idx=66). Local temperature was defined as the average temperature for one year including four distinct seasons: spring, summer, autumn, and winter.

### Definition of incidence and mortality rate

Incidence rate was defined as age adjusted incidence rate (per 100,000) for both bladder and prostate cancers. For prostate cancer, only male data were used. Two forms of mortality rates were used in this study: observed mortality rate (per 100,000) for each county unit, and age standardized mortality rate (ASMR). The observed mortality rate was defined as the number of deaths per 100,000 persons. ASMR was calculated with the following equation: ASMR = sum of (standard population in a specific age group × observed mortality rate in specific age group)/sum of standard population in specific age group. We made two subsets of ASMR: ASMR1 and ASMR2. ASMR1 was calculated by using age groups of 10–19, 20–29, 30–39, 40–49, 50–59, 60–69, 70–79, 80–89, 90–99, and ≥ 100. ASMR2 was calculated by using age groups of < 40, 40–49, 50–59, 60–69, 70–79, 80–89, and ≥ 90.

### Main outcomes and statistical analysis

Basic characteristics of the two geographic groups, including annual incidence, mortality rate, doctor density, urologist density, number of teaching hospitals, annual average income, and annual average temperature were analyzed using *t*-test. Annual changes in doctor density and urologist density were analyzed using linear regression analysis. In this analysis, doctor density and urologist density were fitted to the model with fixed independent variables of county unit (area) after adjusting for years. Trends of incidence and mortality of bladder and prostate cancer were analyzed separately according to county unit using linear regression analysis. In this analysis, dependent variables were incidence of both cancers and ASMR1 and ASMR2 of both cancers. To estimate the role of doctor density and urologist density as risk factors, univariate analyses were performed with independent variables (including numbers of teaching hospitals, annual average income, and annual average temperature) and dependent variables (including incidence of both cancers and ASMR1 and ASMR2 of both cancers). The year was adjusted during these analyses. Multivariable analysis was conducted using significant independent factors identified from univariate analysis.
